# Choroidal and Retinal Abnormalities by Optical Coherence Tomography in Endogenous Cushing’s Syndrome

**DOI:** 10.3389/fendo.2016.00154

**Published:** 2016-12-09

**Authors:** Maria Fernanda Abalem, Marcio Carlos Machado, Helen Nazareth Veloso Dos Santos, Rafael Garcia, John Helal, Pedro Carlos Carricondo, Sérgio Luis Gianotti Pimentel, Mario Luiz Ribeiro Monteiro, Cynthia X. Qian, Marcello Delano Bronstein, Maria Cândida Villares Barisson Fragoso

**Affiliations:** ^1^Department of Ophthalmology and Otolaryngology, University of São Paulo Medical School, São Paulo, São Paulo, Brazil; ^2^Department of Ophthalmology and Visual Sciences, Kellogg Eye Center, University of Michigan, Ann Arbor, MI, USA; ^3^Department of Endocrinology, University of São Paulo Medical School, São Paulo, São Paulo, Brazil; ^4^Endocrinology Service, AC Camargo Cancer Center, São Paulo, São Paulo, Brazil; ^5^Department of Ophthalmology, Maisonneuve-Rosemont Hospital Research Centre, University of Montreal, Montreal, QC, Canada

**Keywords:** hypercortisolism, Cushing syndrome, choroid, optical coherence tomography, eye

## Abstract

**Context:**

Cortisol has been suggested as a risk factor for choroidal thickening, which may lead to retinal changes.

**Objective:**

To compare choroidal thickness measurements using optical coherence tomography (OCT) in patients with endogenous active Cushing’s syndrome (CS) and to evaluate the occurrence of retinal abnormalities in the same group of patients.

**Design:**

Cross-sectional study.

**Setting:**

Outpatient clinic.

**Patients:**

Eleven female patients with CS in hypercortisolism state as determined by the presence of at least two abnormal measurements from urinary cortisol 24 h, no suppression of cortisol with low dose dexamethasone suppression test, and nocturnal salivary cortisol levels and 12 healthy controls.

**Methods:**

Choroidal and retinal morphology was assessed using OCT.

**Main outcome measures:**

Choroidal thickness measurements and the presence of retinal changes.

**Results:**

The mean subfoveal choroidal thickness was 372.96 ± 73.14 µm in the patients with CS and 255.63 ± 50.70 µm in the control group (*p* < 0.001). One patient (9.09%) presented with central serous chorioretinopathy and one patient (9.09%) with pachychoroid pigment epitheliopathy.

**Conclusion:**

Choroidal thickness is increased in the eyes of patients with active CS compared to healthy and matched control. Also, 18.18% of patients presented with macular changes, possibly secondary to choroidal thickening. While further studies are necessary to confirm our findings, excess corticosteroid levels seem to have a significant effect on the choroid and might be associated with secondary retinal diseases.

## Introduction

Endogenous Cushing’s syndrome (CS) is a prototype of metabolic syndrome due to chronic exposure to excess cortisol. The clinical presentation varies depending mostly on the time span the patient is submitted to excessive cortisol production and is characterized by weight gain, facial plethora, central obesity, proximal muscle weakness, hypertension, diabetes mellitus, osteoporosis, neuropsychological disturbances, and purple striae (1).

Several previous reports have documented visual loss in endogenous CS resulting from central serous chorioretinopathy (CSC), a disease that impairs central vision and is characterized by serous retinal detachment, retinal pigment epithelium (RPE) changes, and choroidal vascular hyperpermeability (2–4). While the pathogenesis of CSC is still poorly understood, several risk factors seem to be involved such as younger age, male gender, type-A behavioral pattern, history of organ transplant, pregnancy, systemic blood hypertension, use of exogenous corticosteroids, and endogenous hypercortisolism (5–7).

The mechanisms by which corticosteroids are implicated in the development of CSC are unclear. Currently, it is believed that cortisol excess may lead to a number of choroid abnormalities including (a) increased capillary fragility, leading to choroidal circulation imbalance and leakage of fluid to the subretinal space; (b) blood coagulation in the choroid, leading to choroidal hypoperfusion; (c) inhibition of collagen formation affecting the main component of Bruch’s membrane, the innermost choroid layer; (d) impact on ion and water transport of epithelial cells through interference on mineralocorticoid receptors; and (e) induce systemic hypertension, which in itself is a risk factor for CSC (7).

The choroid is primarily a vascular tissue, which provides the major blood supply to the retina. It can be assessed by two different methods: indocyanine green angiography (ICG), which is a somewhat invasive method, and by enhanced depth image optical coherence tomography (EDI-OCT), a non-invasive exam. Patients with CSC often present choroid vascular hyperpermeability on ICG and increased choroidal thickness when measured by EDI-OCT (8–11).

Several reports have reported increased choroidal thickness in both affected and fellow eyes of CSC patients, compared to control subjects. Moreover, in cases of unilateral CSC, the affected eye seems to be thicker than the fellow eye (12–14).

Because of the previous association of CSC both with excess corticosteroid levels and choroidal thickening, we hypothesized that patients with endogenous hypercortisolism might develop choroidal thickening that could be a predisposing factor for CSC and other diseases possibly associated to choroidal thickening such as pachychoroid pigment epitheliopathy (PPE), pachychoroid neovasculopathy (PN), and polypoidal choroidal vasculopathy (PCV) (15, 16).

More recently, Karaca et al. reported a thicker choroid in patients with CS possibly related to increased levels of ACTH itself, increased levels of plasma cortisol, or both (17). Therefore, the aim of the present study was to evaluate possible effects of excess corticosteroids exposure on choroidal thickness using OCT and to investigate possible retinal abnormalities in patients with active hypercortisolism due to endogenous CS.

## Materials and Methods

This cross-sectional study was performed at the Division of Ophthalmology and the Department of Endocrinology of the University of São Paulo Medical School. The study was approved by our institutional review board and conducted according to the Declaration of Helsinki. Informed written consent was obtained from all participants.

A diagnosis of active CS was established by the presence of at least two unequivocal altered of first line screening for CS [urinary cortisol (UC) 24 h, nocturnal serum, or salivary cortisol] and serum cortisol (Fs) after low dose dexamethasone suppression test (*F*s > 1.8 μg/dL or 50 mmol/L). Eleven patients were selected for the study, nine due to ACTH-dependent and two due to ACTH-independent CS. Twelve healthy patients were recruited as a control group, matched for age and gender to the patients. Control patients with systemic diseases, like arterial systemic hypertension, diabetes, autoimmune diseases, or any other condition that could affect the choroid and retina were excluded. Moreover, those undertaking exogenous steroids in either anti-inflammatory or immunosuppressive dose in the past 12 months were also excluded.

Considering that CSC can be present unilaterally, both eyes were included. Exclusion criterion for patients were the presence of refractive error exceeding ±6.00 diopters (D) of spherical equivalent, presence of more than two diopters of keratometric astigmatism, axial length of more than 26.5 mm, important media opacity (resulting in poor OCT image quality), history of uveitis and retinal diseases other than CSC, and any choroidal-related diseases, such as PPE, PN, and PCV. Patients with a history of glaucoma or any other optic neuropathy, intraocular surgery in the past 3 months (including cataract surgery), intravitreal injections (steroids and/or anti-vascular endothelial growth factor), and/or laser treatment were also excluded.

### Patient Assessment

All patients were submitted to a comprehensive ocular examinations, including biomicroscopy of anterior and posterior segments, indirect fundoscopy, axial length measurement (IOL Master, Carl Zeiss, Jena, Germany), and spectral-domain optical coherence tomography (SD-OCT) (Spectralis; Heidelberg Engineering, Heidelberg, Germany) with an EDI protocol (horizontal and vertical scans, 7 sections, high resolution mode, 25 frames). SD-OCT images were routinely obtained at the same time, avoiding diurnal variations of choroidal vasculature. Two raters experienced in examining EDI-OCT images (Maria Fernanda Abalem and Helen Nazareth Veloso Dos Santos) independently evaluated the choroidal features. The third rater (Pedro Carlos Carricondo) was consulted when the two raters disagreed.

### Choroidal Evaluation

The following features were addressed, as described previously (10, 18):

(A) Total choroidal thickness (TCT) was measured at the subfoveal position. The measures were performed manually using the caliper of the Eye Explorer software (v. 6.0.9.0; Heidelberg Engineering) with 80% zoom. The measure was obtained perpendicularly, from the outer edge of the hyperreflective RPE to the hyperreflective inner sclera.
(B) Choroidal segmentation: three large choroidal vessels measuring at least 100 μ in diameter, located subfoveal, and a perpendicular line from the innermost point of each one was drawn so these lines would intersect with the lines used to measure TCT. The Haller layer was determined by measuring perpendicularly from the inner border of the sclera to the innermost line of the selected large choroidal vessels. The remaining distance of choroidal thickness was considered as the choriocapillaris/Sattler layer complex. These two layers were analyzed together because current OCT technology still does not allow for the separation of both layers.

### Retinal Evaluation

The main characteristics of CSC, PPE, PN, and PCV were addressed, including the presence of subretinal fluid, intraretinal cysts, ellipsoid layer disruption, drusen, pigmented epithelium detachment, and choroidal neovascularization.

### Statistical Analysis

All statistical analyses were performed using the SPSS software version 18.0 (SPSS, Chicago, IL, USA). Numerical values were reported as mean ± SD. Choroidal thickness parameters, including the thickness of Haller layer, the choriocapillaris/Sattler layer were estimated and compared. The interobserver reliability was expressed as the intraclass correlation coefficient. Also, *p* values of 0.05 were considered statistically significant.

Optical coherence tomography measurements of both eyes were compared by using Generalized Estimating Equation (GEE) models, in order to compensate inter-eye dependencies. In this study, all patients and controls had both eyes included. As eyes of the same individual were expected to have some degree of intercorrelation with respect to OCT parameters, GEE models were used to adjust for within-patient inter-eye correlations. GEE models are generalized linear models that allow for the specification of within-group correlations when evaluating the ability of one or several independent variables to predict a dependent variable.

## Results

From 2010 to 2015, a total of 11 patients with active endogenous CS, mean age of 38.45 years (±16.32), and 12 control patients, mean age of 51.33 years (±16.64), were included in this study. By chance, all of them were female subjects. There was no statistical difference between age of patients and controls (*p* = 0.08). The etiologies of CS were eight patients with (72.7%) Cushing’s disease (pituitary adenoma), one (9.1%) with adrenocotical adenoma, one (9.1%) with adrenocortical carcinoma, and one (9.1%) with primary macronodular adrenal hyperplasia (PMAH). None of our patients had pituitary tumor with suprasellar extension as confirmed by magnetic resonance imaging. As required to be eligible, all patients were in hypercortisolism state according to UC 24 h levels and nocturnal salivary cortisol (patient number 9) (Table 1). No control patient presented with any autoimmune and/or vascular systemic and ocular condition as required to be included in this study. However, one patient had prior history of breast cancer diagnosed and surgically treated 10 years ago, and two patients had prior history of cholecystectomy in the past 5 years due to chronic cholecystitis. Any patient had history of exogenous steroids intake in the past 12 months (Table 2).

**Table 1:** **Clinical and hormonal data of Cushing’s syndrome patients**.

*N*	Patient	Age (years old)	Gender	UC 24 h (μg/24 h)	ACTH (pg/mL)	Etiology	Pituitary MRI	Active hypercortisolism state (months)
1	APS	33	Female	837^a^	126.2	CD	MICRO	115
2	DPM	22	Female	502^a^	42.5	CD	MACRO	28
3	FSF	32	Female	399^b^	126.8	CD	MACRO	90
4	LLA	35	Female	1288^a^	42.2	CD	MICRO	5
5	MLP	51	Female	826^a^	42.2	CD	MACRO	38
6	MISO	45	Female	1485^a^	87.1	CD	MICRO	15
7	PRM	29	Female	371^a^	33.2	CD	MICRO	80
8	SAGP	26	Female	334^b^	77.5	CD	MACRO	18
9	VAS	69	Female	295^a^/0.8^c^	<2.0	CS	–	360
						PMAH		
10	GMS	19	Female	982^a^	<2.0	CS	–	48
						Adenoma		
11	VLCL	62	Female	733^a^	<2.0	CS	–	12
						Carcinoma		

*UC, urinary cortisol 24 h (normal range: ^a^Reference: 50–310 μg/24 h or ^b^Reference: 3–43 μg/24 h); ^c^Midnight salivary cortisol (normal value: up to 0.12 μg/dL); ACTH (normal range: 7.2–63.30 pg/mL); CD, Cushing’s disease; CS, Cushing’s syndrome; PMAH, primary macronodular adrenal hyperplasia; MRI, magnetic resonance imaging; MICRO (microadenoma <10 mm); MACRO (macroadenoma ≥10 mm) based on MRI*.

**Table 2:** **Demographical and clinical data of control patients**.

*N*	Patient	Age (years)	Gender	Comorbidities
1	MEGB	27	Female	No
2	APP	27	Female	No
3	MS	29	Female	No
4	CSS	43	Female	No
5	NAO	51	Female	No
6	ESL	54	Female	Cholescystitis^a^
7	DMC	55	Female	Breast cancer^a^
8	HHR	60	Female	No
9	MHGK	60	Female	Cholescystitis^a^
10	ESG	65	Female	No
11	VB	68	Female	No
12	ANB	77	Female	No

*^a^Surgically treated*.

The interobserver reliability of the two retinal specialists who performed the measurements was very good. The intraclass correlation coefficients were of 0.876 for TCT, 0.864 for the thickness of Haller layer, and 0.996 for the choriocapillaris/Sattler layer.

**Figure 1:**
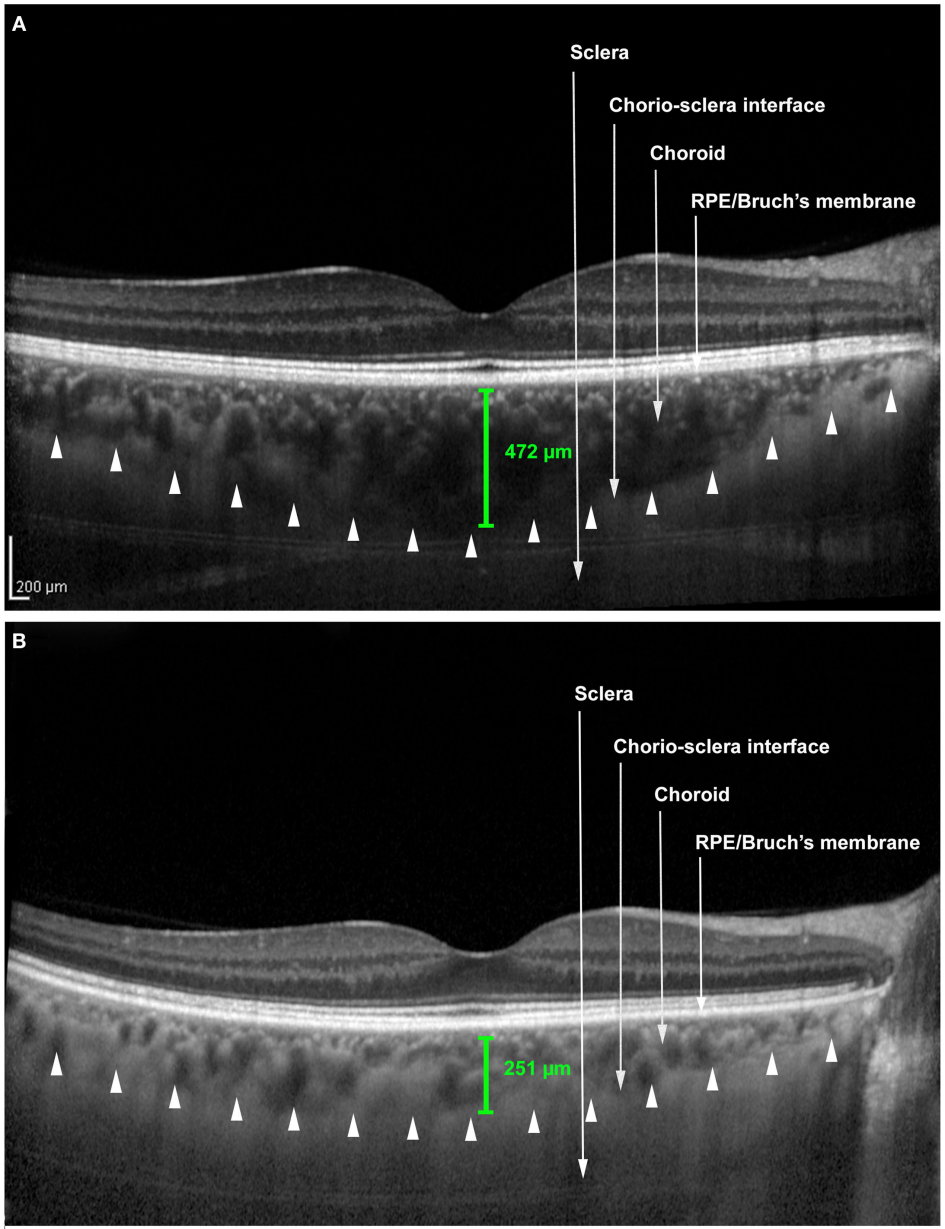
**Enhanced depth image optical coherence tomography scans**. **(A)** Patient with Cushing’s syndrome. **(B)** Control patient. The caliper (green) represents the choroidal thickness. The arrowheads represent the anterior boundary of the sclera.

The mean subfoveal choroidal thickness was 372.96 ± 73.14 µm in eyes of patients with CS and 255.63 ± 50.70 µm in eyes of the control group, with statistically significant difference (*p* < 0.001, GEE) (Figure 1). The results were similar in the mean thickness of Haller layer, measuring 342.05 ± 73.77 µm in eyes of patients with CS and 231.92 ± 51.54 µm in eyes of the control group (*p* < 0.001, GEE). The mean thickness of choriocapillaris/Sattler layer also was statistically different, measuring 34.91 ± 22.36 µm in the group with CS and 23.71 ± 14.93 µm in the control group (*p* < 0.001, GEE).

**Figure 2:**
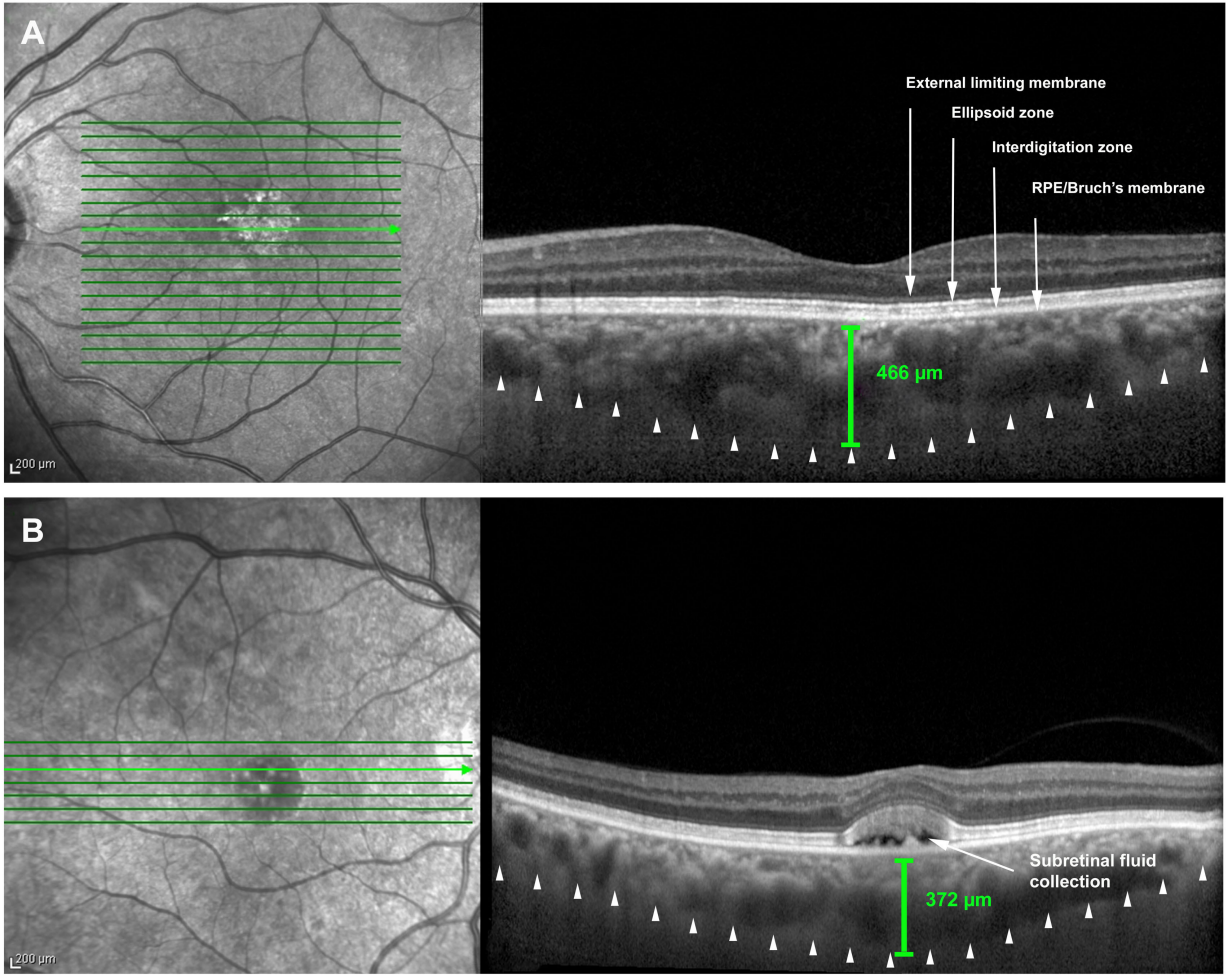
**Macular optical coherence tomography scans**. **(A)** Left eye of patient 1 with drusen-like lesions, associated with ellipsoid zone irregularity and choroidal thickening. **(B)** Right eye of patient 10 with subretinal fluid (arrow), associated to ellipsoid zone irregularity and choroidal thickening.

Two patients (18.18%) presented with macular changes. Patient 1 presented drusen-like lesions, associated with ellipsoid zone irregularity on both eyes, suggesting PPE. Patient 10 presented with bilateral subretinal fluid, associated to ellipsoid zone irregularity and drusen-like lesions, suggestive of CSC (Figure 2).

## Discussion

Cushing’s syndrome is a medical condition that can be associated with numerous deleterious changes in various organ systems leading to comorbidities and increased mortality ratio. This study addressed possible effects of excessive corticosteroid levels on the choroid in patients with active hypercortisolism due to endogenous CS. We have found that TCT was significantly greater in patients with endogenous CS, when compared with age- and gender-matched controls, without any history of systemic and ocular vascular or autoimmune condition that could directly or indirectly affect both retina and choroid as well as prior history of exogenous steroids intake in the past 12 months.

Our findings are in agreement with a number of previous studies, which demonstrated a thicker choroid in patients with CSC, a condition that is also related to elevated systemic levels of corticosteroids. Several reports identified corticosteroids as a significant risk factor for the development of acute, exudative macular manifestation in patients with CSC, either by endogenous Cushing syndrome or by exogenous intake (2, 7, 19, 20). Garg et al. also demonstrated an increase in the 8:00 a.m. and 11:00 p.m. serum cortisol and UC 24 h levels in acute CSC patients compared to healthy subjects (21, 22).

Additionally, other endocrine abnormalities were found in a case series of 24 patients with CSC, such as UC 24 h and tetrahydroaldosterone levels, low serum aldosterone levels, elevated single morning plasma catecholamine level, but normal 24-h urine metanephrines (23). The current study is partially in accordance with a recent study by Karaca et al. that encountered an association between choroidal thickening and ACTH levels in patients with chronic CS, suggesting that ACTH itself, increased cortisol levels, or both might be related to this finding (17). However, in this study it is not clear about the cortisol state of patients because only the midnight and morning serum cortisol were evaluated. It is possible that these patients had only absence of circadian secretion due to medical treatment (e.g., cabergoline, ketoconazole, or pasireotide) in the presence of normal levels of UC 24 h (24). The total cortisol secretion measured by UC 24 h should have been demonstrated to characterize hypercortisolism activity. Moreover, to the best of our knowledge, there are no melanocortin receptors on the choroid; hence, it seems more likely that increased cortisol levels may be responsible for the choroidal changes.

The choroid can change their volume, thereby their thickness as much as fourfold over a few days. There are five possible mechanisms underlying choroidal thickening. The most likely mechanism is due to the synthesis of osmotically active molecules that act as “sponges” leading to expansion of the lacunae (25). Another mechanism refers to changes in vascular permeability. An increase in capillary permeability may allow proteins to move into the extracellular matrix and/or lymphatic, followed by passive fluid flow (26). A third mechanism proposed derives from the aqueous humor flow from the anterior chamber to the choroid. Since the choroid is part of the uveoscleral outflow pathway, an increase in the humor aqueous flow may be transmitted to the choroid and expand it (25, 27). Another similar mechanism is by moving fluid across the RPE. There is a flow of ions and water between the retina and the choroid, and changes in this flow might impact on choroidal thickness (28). Finally, the choroid may thicken because of changes in the tonus of non-vascular smooth muscle cells that are controlled by both sympathetic and parasympathetic inputs. If these cells relax, the choroid may become thicker (29–31).

The excess of cortisol has several effects that may be involved in the mechanisms that modulate choroidal thickness contributing for both thickening and thinning. Corticosteroids may increase osmolarity and active fluid flow into the choroidal vasculature (32); increase renal tubular sodium reabsorption and intravascular volume expansion (33); dilate choroidal endothelial cells (33, 34); and inhibit the formation of collagen, which may facilitate moving fluid across the RPE (7). On the other side, corticosteroids impair vasodilation, induce vasoconstriction (also by enhancement of the renin angiotensin system), and increase the vascular sensitivity to the effects of catecholamines (33). These effects are thought to thin the choroid. We believe the thickening mechanisms have predominated because thinning is somewhat limited by mechanical restrictions and because intravascular expansion may lead to expansion of the lacunae, which is the major anatomical change, related to choroidal thickening (31).

In the current study, the Haller layer thickness was greater in patients with CS than in controls. The choriocapillaris/Sattler layer complex did not show any difference in both groups. These findings suggest that increased TCT was at least in part due to increased Haller layer thickness. Chung et al. also assessed the Haller and Sattler layers in patients with CSC and showed a greater ratio of Haller layer/TCT. They believed these findings were possibly due to the higher number of non-vascular smooth muscle cells in the Haller layer, which may stretch out, leading to vascular dilation and accumulation of fluid in the stroma. Alternatively, the lower number of these cells in the choriocapillaris/Sattler layers might keep these layers relatively stable under sympathetic stimuli (10). Furthermore, the lacunae are more predominant and larger in the suprachoroid, which may explain a greater vascular expansion in this location (31).

Han et al. prospectively evaluate the effects of corticosteroids on choroidal thickness in patients who required high-dose corticosteroid pulse therapy for a short period, with a 1-month follow-up (35). While the authors did not find changes in choroidal thickness, they described some features of CSC like pigment epithelial detachment and subretinal fluid in one patient who also presented with a thicker choroid. This lack of consistent changes might be explained by the fact that these patients were under high levels of cortisol, but for a short period. In our study, all patients were under prolonged and excessive cortisol exposure, suggesting that choroidal thickening also depend on both factors: degree and duration of hypercortisolism, as occurs with systemic manifestations (35).

We also found one patient (9.09%) with a PPE and another (9.09%) with bilateral CSC, conditions that were demonstrated to be associated with choroidal thickening (10, 15). The frequency of PPE in overall population is still unknown. It is a misdiagnosed condition, in which there is a marked disturbance in the RPE cells with possible visual impairment (15). According to Bouzas et al., the incidence of CSC is 5% in patients with endogenous CS. In this study, 3 out of 46 women and 1 out of 14 men were affected (2). We believe this condition might be underestimated in patients with CS, because (1) during the hypercortisolism state, the patient and the physician might not note this kind of symptom and (2) CSC is self-limited in most cases and visual acuity usually returns to 20/25 or better (36, 37), although chronic and atypical cases may also occur in 30–50% of cases (7, 38, 39). Thus, we believe patients with CS might require proper retinal evaluation.

To our knowledge, this is the first study to describe choroidal and retinal abnormalities in patients with active endogenous CS-dependent and independent of ACTH during hypercortisolism state, based on high levels of UC 24 h, except in the patient with PMAH, where cortisol activity was characterized based on both midnight salivary cortisol and low dose dexamethasone suppression test. As increased cortisol activity is the hallmark of CS, this specific group of patients provides better understanding of the effects of cortisol on the choroid. We believe the increased osmolarity, the mineralocorticoid effects of cortisol, and the activation of the renin angiotensin system in patients with CS may be involved in the pathophysiology of choroidal expansion explaining the increased choroidal thickness measurements.

The current study has a number of limitations including the cross-sectional design and the small number of patients. However, our data serve to emphasize the existence of an additional morbidity in patients with endogenous CS. Further comparative studies are therefore required to better evaluate (1) the role of other adrenal hormones, such as catecholamines that also seem to be involved in the pathogenesis of CSC (40, 41); (2) the long-term effects of cortisol on both the choroid and the retina of these patients; and (3) the visual impairment caused by such abnormalities. Since choroidal thickening is associated to retinal conditions, like CSC, PPE, PN, and PCV, patients with CS in hypercortisolism state should be evaluated for these conditions.

## Ethics Statement

Comissão de Ética para Análise de Projetos de Pesquisa do Hospital das Clinicas da Faculdade de Medicina da Universidade de São Paulo. This is a non-interventional study. The patients underwent routine ophthalmic evaluation and optical coherence tomography. The latter does not require any additional procedure and/or preparing. It is a non-invasive imaging method. The patients were told about the minimum risk of participating in this study and also about the possibility of being treated in case of eye-disease finding.

## Author Contributions

MA: design, data collection, data analysis, and writing; MCM: design, data analysis, and reviewing; HS and RG: data collection; PC: design, statistical analysis, data analysis, and reviewing; JH: design, data analysis, and reviewing; SP: reviewing; CQ: data collection and figures edition; MB: design and reviewing; MF: design, data collection, data analysis, and reviewing.

## Conflict of Interest Statement

The authors declare that the research was conducted in the absence of any commercial or financial relationships that could be construed as a potential conflict of interest. The handling Editor declared a shared affiliation, though no other collaboration, with the author CQ and states that the process nevertheless met the standards of a fair and objective review.
